# Hematopoietic stem cell transplantation activity in China 2019: a report from the Chinese Blood and Marrow Transplantation Registry Group

**DOI:** 10.1038/s41409-021-01431-6

**Published:** 2021-08-25

**Authors:** Lan-Ping Xu, Pei-Hua Lu, De-Pei Wu, Zi-Min Sun, Qi-Fa Liu, Ming-Zhe Han, Xi Zhang, Yong-Ping Song, Xian-Min Song, Jian-da Hu, He Huang, Yong-Rong Lai, Ding-Ming Wan, Jing Chen, Chun-Fu Li, Ling-Hui Xia, Jing-Bo Wang, Dai-Hong Liu, Xiao-Jun Huang, on behalf of the Chinese Blood and Marrow Transplantation Registry Group

**Affiliations:** 1 Peking University People’s Hospital, Peking University Institute of Hematology, National Clinical Research Center for Hematologic Disease, Beijing Key Laboratory of Hematopoietic Stem Cell Transplantation, Beijing, China; 2 Hebei Yanda Lu Daopei Hospital & Beijing Ludaopei Hospital, Langfang, Hebei & Beijing China; 3 The First Affiliated Hospital of Soochow University, National Clinical Research Center for Hematologic Disease, Soochow, China; 4 Anhui Provincial Hospital, Hefei, China; 5 Nanfang Hospital, Guangzhou, China; 6 Blood Diseases hospital, Chinese Academy of Medical Sciences, Tianjin, China; 7 Xinqiao Hospital Army Medical University, Chongqing, China; 8 Henan Cancer Hospital, the Affiliated Cancer Hospital of Zhengzhou University, Zhengzhou, China; 9 Shanghai General Hospital, Shanghai, China; 10 Fujian Medical University Union Hospital, Fujian Provincial Key Laboratory of Hematology, Fujian Institute of Hematology, Fuzhou, China; 11 The First Affiliated Hospital, Zhejiang University, Hangzhou, China; 12 The First Affiliated Hospital of Guangxi Medical University, Nanning, China; 13 The First Affiliated Hospital of Zhengzhou University, Zhengzhou, China; 14 Shanghai Children’s Medical Center, Shanghai, China; 15 Nanfang-Chunfu Children’s Institute of Hematology & Oncology Taixin Hospital, Dongguan, China; 16 Wuhan Union Hospital, Wuhan, China; 17 Aerospace Center Hospital, Beijing, China; 18 Chinese PLA General Hospital, Beijing, China

**Keywords:** Stem-cell therapies, Epidemiology

## Abstract

Between 2008 and 2019, 58,914 hematopoietic stem cell transplantations (HSCTs) were reported to the Chinese Blood and Marrow Transplantation Registry Group (CBMTRG) throughout China. In this report, we focus on 2019 data and describe current trends in HSCT in China. There was continued growth in transplant activity in China, with a rapid increase in haploidentical HSCT. In 2019, a total of 12,323 cases of HSCT were reported from 149 transplant teams, 78% (9597 cases) were allogeneic HSCTs. Haploidentical donor (HID) HSCT accounted for 60% (5771 cases) of allogeneic HSCT. The most common indications for allogeneic HSCT for malignant disease were acute myeloid leukemia (AML) (37%) and acute lymphoblastic leukemia (ALL) (24%), and the largest proportion of non-malignant diseases comprised aplastic anemia (AA) (13%). Multiple stem cell source composed 70% of HID and 28% of MSD, which was typical in China. The BuCy based regimen (59%) was the most popular conditioning regimen for allogeneic HSCT, followed by the BuFlu based regimen (23%) and TBI-based regimen (12%). This survey clearly shows comprehensive information about the current state and recent trends for HSCT in China. Further efforts should be made to obtain detailed information.

## Introduction

Hematopoietic stem cell transplantation (HSCT) is an established treatment for many congenital or acquired disorders of the hematopoietic system and some other life-threatening diseases [1–4]. According to surveys of transplantation activity from the Worldwide Network for Blood and Marrow Transplantation Group, the Center for International Blood and Marrow Transplant Research (CIBMTR), the European Group for Blood and Marrow Transplantation (EBMT), and the Asia-Pacific Blood and Marrow Transplantation Group (APBMT), the annual global frequency of HSCT has been increasing steadily and regional differences in donor type, stem cell sources and indications have been confirmed [5–11]. The Chinese Blood and Marrow Transplantation Registry Group (CBMTRG) was established in 2007 and collect transplant data in China every 6 months. In 2017, the CBMTRG published a report of transplant activity during 2008–2016 in China, which demonstrated a continuous increase in HSCT, especially in HID [9]. In the last year, the annual number of HSCTs in China more than 10,000 for the first time, and 60% of allogeneic HSCTs were HID. The number of HID HSCT first exceeded 5000 per year, which is much higher than that in the US or Europe (1769 [9] and 3538 [10] in 2019). With the intention of providing useful information for health professionals all around the world, this cross-sectional analysis was performed based on the CBMTRG survey data of 2019, offering an overview of recent trends in HSCT in China.

## Materials and methods

### Data collection

A nationwide registration of HSCT has been organized by the Stem Cell Application Section of the Hematology Branch of the Chinese Medical Association since 2008. Data including transplant numbers, patient gender and age, disease, donor type, stem cell source, conditioning regimen, and protocols for graft-versus-host disease (GVHD) prophylaxis were reported from participating teams to the CMBTRG by Excel form every 6 months. Data were collected by patient level, and first transplant or multiple transplant for a patient was not distinguished. This was a retrospective study focusing on data collected from HSCT centers in China in 2019. The teams involved in this study are listed in the Supplementary Appendix in alphabetical order by the name of the hospital.

### Results participating teams

A total of 149 transplant teams participated in the 2019 survey, these teams were located in 27 provinces, municipalities or autonomous regions, among which the Beijing area contributed the largest number of transplants (2620, 21%), followed by Guangdong Province (1592, 13%) and Jiangsu Province (1108, 9%). A total of 126 teams (85%) performed both allogeneic and auto HSCTs. The transplant activity was restricted to auto HSCT in 9 teams (6%) and to allogeneic HSCT in 14 teams (9%). HID HSCT was reported by 135 teams (91%). 116 teams reported both adult and pediatric transplants, 23 teams reported only adult transplants, and 10 teams reported only pediatric transplants.

The median number of transplants is 42 (1–1014). Three teams reported >500 HSCTs: Peking University People’s Hospital, Peking University Institute of Hematology (1014), The First Affiliated Hospital of Soochow University (762), and Hebei Yanda Lu Daopei Hospital (758). For allogeneic transplants, the median number was 29 (1–944), and for autologous transplants the median number was 13 (1–147). Data are shown in Table 1. It is obvious that large teams (>100 HSCT per year) performed more than half of all the transplants, and 80% of the teams were in small (<25 HSCT per year) or medium (25–100 HSCT per year) in size. Compared with those in 2016, team numbers of all the 3 groups increased. The median number of HSCTs per team remained constant for small and medium teams but increased from 137 to 189 for large teams.

**Table 1:** Numbers of teams and transplants by size category.

Team size	No. of teams		Percentage of total teams		No. of HSCT		Percentage of total transplants		Median (range) HSCT per team	
	2019	2016	2019	2016	2019	2016	2019	2016	2019	2016
HSCT										
<25 HSCT per team	53	21	36	28	700	303	6	5	11 (1–25)	15 (2–24)
25–100 HSCT per team	66	38	44	50	3918	2110	32	36	56 (26–100)	52 (27–99)
>100 HSCT per team	30	17	20	22	7705	3458	62	59	189 (101–1014)	137 (101–785)
Total	149	76			12,323	5871			42 (1–1014)	46 (2–785)
Allogeneic										
<25 HSCT per team	66	26	47	36	628	280	6	6	9 (1–25)	11 (1–25)
25–100 HSCT per team	48	36	34	49	2573	1881	27	40	53 (28–97)	47 (26–98)
>100 HSCT per team	26	11	19	15	6396	2510	67	54	175 (109–944)	153 (106–752)
Total	140	73			9597	4671			29 (1–944)	37 (1–752)
Autologous										
<25 HSCT per team	106	53	78	77	1156	551	42	46	10 (1–25)	11 (1–23)
25–100 HSCT per team	28	16	21	23	1420	649	52	54	48 (26–85)	36 (28–71)
>100 HSCT per team	1	0	1	0	147	0	5	0	147	
Total	135	69			2723	1200			13 (1–147)	13 (1–71)

The 2016 data are based on the teams that participated in both the survey of first half and second half of the year.

### Number of transplants

**Fig. 1:**
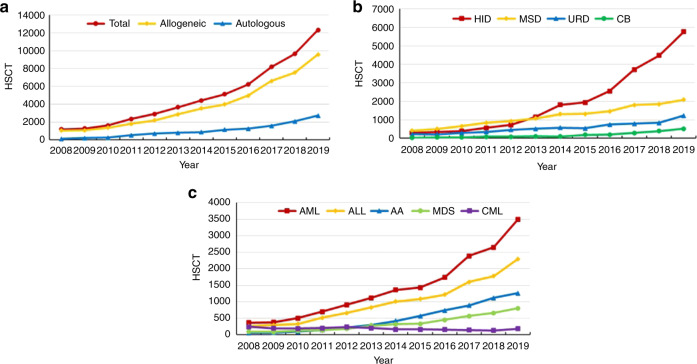
HSCT trend in China during 2008–2019. **a** Annual number of HSCT. **b** Annual number of allogeneic HSCT based on donor type. **c** Annual trend of absolute numbers of disease indications for allogeneic HSCT.

Between 2008 and 2019, 58,914 HSCTs were reported, with 46,619 (79%) allogeneic. The annual number of transplants reached more than 10000 for the first time in 2019, counting as 12,323 HSCTs, of which 2723 (22%) were autologous, 9597 (78%) were allogeneic, and 3 were syngeneic. Among allogeneic HSCT, HID accounted for the highest proportion at 60% (5771 cases), increasing by 29% compared with 2018. The proportions of matched sibling donor (MSD), unrelated donor (URD), and cord blood (CB) HCSTs among allogeneic HSCT were 22%, 13%, and 5%, respectively. The trend in HSCT by donor type in the past 12 years is presented in Fig. 1a, b.

**Fig. 2:**
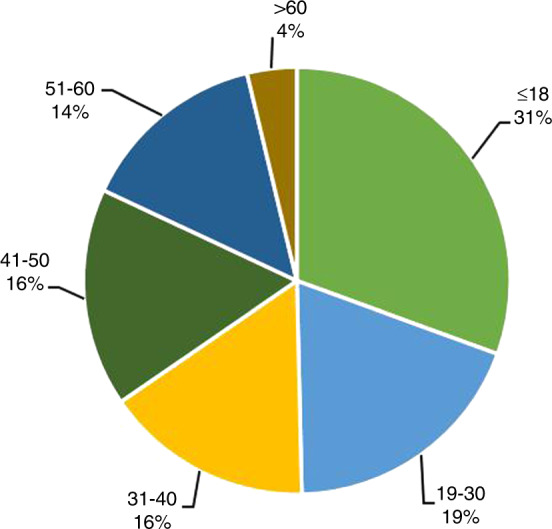
Distribution of ages of HSCT recipients in China 2019. Distribution of ages of HSCT recipients in China 2019.

42% of the HSCT patients in 2019 were female, and 58% were male. The median age of the HSCT patients was 31 (0.2–79) years, and the median ages of auto and allo-HSCT patients were 49 (2–75) and 25 (0.2–79) years, respectively. The number of pediatric patients (≤18 years of age) was 3752, and 96% of them underwent allogeneic HSCT. The number of elderly patients (>50 years of age) was 2213, and 44% of them underwent allogeneic HSCT. There were 461 patients >60 years of age, and 26% of them underwent allogeneic HSCT. The proportion of each age group is shown in Fig. 2.

The transplant rates for allogeneic and autologous HSCT in China were 68.5 and 19.4 per 10 million population, respectively. Among the 27 provinces, municipalities or autonomous regions, the median transplant rates per 10 million population were 32.0 (0–848.6) for allogeneic HSCT and 13.2 (0– 217.7) for autologous HSCT. Beijing had the highest transplant rate for both allogeneic and autologous HSCT (Population numbers were obtained from the official website of the National Bureau of Statistics of China (http://www.stats.gov.cn/)).

#### Disease indication

**Fig. 3:**
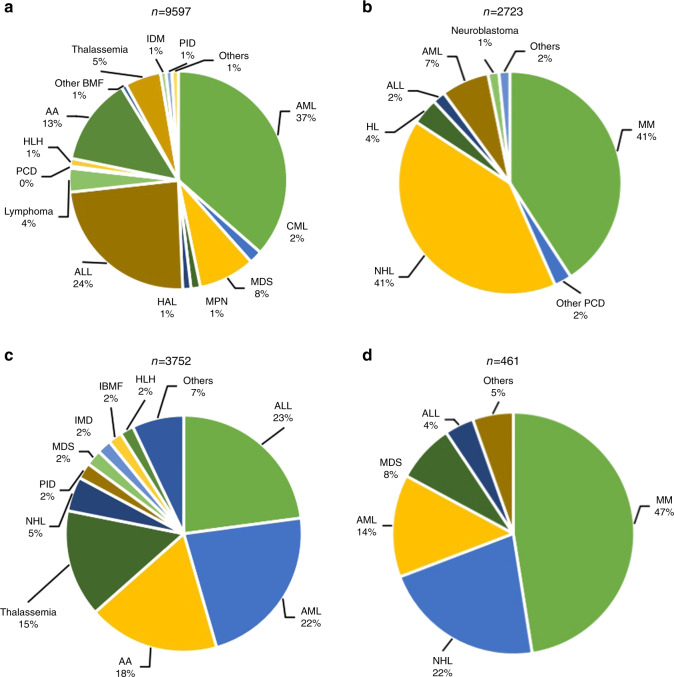
Distribution of disease indications for HSCT in China 2019. **a** Relative proportion of allogeneic HSCT. **b** Relative proportion of autologous HSCT. **c** Proportions of indication for pediatric patients. **d** Proportion of indication for elderly patients.

The distribution of disease indications for allogeneic and autologous HSCT is shown as pie graphs in Fig. 3a and b. Main indications for HSCT were acute leukemia (acute myeloid leukemia (AML), acute lymphoblastic leukemia (ALL), and mixed phenotype acute leukemia), with a total of 6121 cases (96% allogeneic HSCT and 4% autologous HSCT). The predominant indications for allogeneic HSCT were AML (3500 cases, 37%) and ALL (2294 cases, 24%). Additionally, aplastic anemia (AA) (1252 cases, 13%), myelodysplastic syndrome (MDS) (802 cases, 8%), Thalassemia (512 cases, 5%), lymphoma (338 cases, 4%), chronic myelogenous leukemia (CML) (185 cases, 2%) were identified. Regarding autologous HSCT, multiple myeloma (MM) and non-Hodgkin’s lymphoma (NHL) were the most common indications, both with proportions of 41%. The proportions of indications for allogeneic and autologous HSCT are shown in Fig. 3a, b. The trend in the numbers of main indications for allogeneic HSCT is shown in Fig. 1c. Except for CML, all the diseases showed steady growth.

The indications and donor type are shown in Table 2. In 2019, the number of AML patients who underwent HSCT increased by 30% compared to the number in 2018. Among the 3687 cases of AML, 60% (2219 cases) received stem cells from HIDs, and the remaining cases received stem cells from MSDs (21%, 761 cases), URDs (10%, 362 cases), or CB (4%, 158 cases), or via autologous transplantation (5%, 187 cases). Similar to AML, among 2346 ALL patients, which was 28% higher than the number in 2018, HID HSCT accounted for 65% (1516 cases), and MSD, URD, CB, and autologous HSCT accounted for 19% (451 cases), 10% (229 cases), 4% (98 cases), and 2% (50 cases), respectively. CML cases had decreased slowly since 2012 but increased by 51 cases in 2019, yet the proportion still dropped to only 2% of allogeneic HSCT. Among the 185 CML patients, 120 (65%) received HID HSCT. In addition, 478 of 807 (59%) MDS patients chose HID HSCT, more often than other donor types, including MSD (209 cases, 26%), URD (88 cases, 11%), cord blood (27 cases, 3%), and autologous HSCT (5 cases).

**Table 2:** Numbers of HSCT in China 2019 by indication, donor type, and stem cell source.

	MSD								HID								URD^a^	CB	Syngeneic	Auto^a^	Total
	PB		BM		BM + PB		Unknown	Total	PB		BM		BM + PB		Unknown	Total					
	PB only	PB + third-party CB	BM only	BM + third-party CB	BM + PB only	BM + PB + third-party CB			PB only	PB + third-party CB	BM only	BM + third-party CB	BM + PB only	BM + PB + third-party CB							
Myeloid malignancies	715	13	1	0	183	4	125	1041	820	112	24	2	1430	134	374	2896	485	196	0	193	4811
AML	536	10			129	3	83	761	642	78	11	2	1113	100	273	2219	361	158		188	3687
CML	28				6		10	44	37	3			59	3	18	120	16	5		0	185
MDS	135	3	1		41	1	28	209	129	13	13		232	29	62	478	88	27		5	807
MDS/MPN	12				4		3	19	7	16			19	2	21	65	19	5			108
MPN	4				3		1	8	5	2			7			14	1	1			24
Lymphoid malignancies	372	5	0	6	109	3	40	535	425	67	4	8	970	77	194	1745	271	107	2	2449	5109
ALL	312	2		6	96	2	33	451	352	56	4	7	871	66	160	1516	229	98	2	50	2346
MM	6				4	1	1	12	12							12		0		1112	1136
other plasma cell disorders	2							2	1	1						2	1			69	74
HL	2							2					1			1	1	0		106	110
NHL	50	3			9		6	68	60	10		1	98	11	34	214	40	9		1112	1443
Mixed phenotype acute leukemia	13				4		5	22	18	3	1		41	9	11	83	7	5		1	118
Solid tumors	0	0	0	0	0	0	0	0	0	1	0	0	1	0	0	2	0	0	0	50	52
Neuroblastoma										1						1				43	44
other solid tumors													1			1				7	8
Nonmalignant disorders	206	6	6	21	143	3	79	464	218	66	16	3	494	68	120	985	445	194	1	1	2090
SAA	124	2	1		92	2	50	271	130	23	14		380	28	73	648	208	125	1	0	1253
Other bone marrow failure	6						2	8					18	1	2	21	27	8			64
Thalassemia	53	3	4	21	49	1	25	156	50	38	2	3	44	19	31	187	165	4		0	512
Inherited disorders of metabolism	3							3	6	1			6	6	2	21	13	31			68
Primary immune deficiencies	8		1				1	10	4	3			8	14	1	30	18	23			81
Hemophagocytic lymphohistiocytosis	12	1			2		1	16	28	1			38		11	78	14	3		1	112
others	15				2		4	21	16	1			36	3	4	60	21	12		29	143
Total	1321	24	7	27	441	10	253	2083	1497	250	45	13	2972	291	703	5771	1229	514	3	2723	12,323

Not all centers reported stem cell source data, and that is classified as unknown.

^a^97% of URD and 99% of autologous HSCT used PB as stem cell source, so total number of URD and autologous HSCT are shown in the table.

**Fig. 4:**
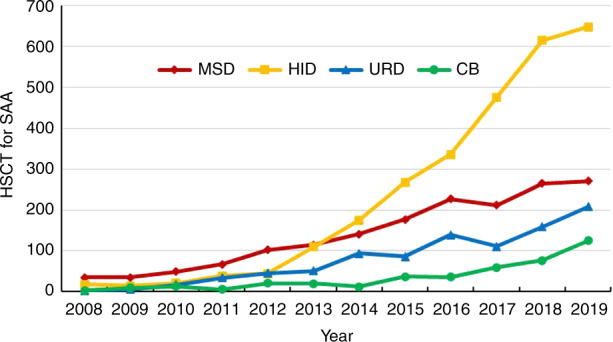
Annual trend of absolute number of HSCT for severe aplastic anemia based on donor type. Annual trend of absolute number of HSCT for severe aplastic anemia based on donor type.

Regarding non-malignant indications, AA and thalassemia are cardinal diseases. As shown in Fig. 1c, the number of allogeneic HSCT for AA patients continuously increased in CBMTRG database. It has surpassed MDS since 2012 and ranked third in number, after AML and ALL, which can be attributed to the development of HID HSCT to some extent (Fig. 4). Of the 1253 AA patients, 648 (51%) received stem cells from HIDs, followed by MRD (271 cases, 22%), URD (208 cases, 17%), cord blood (125 cases, 10%), and 1 case of syngeneic donor. In terms of the number of cases, thalassemia constituted the second largest group among non-malignant diseases. The proportions of HID, URD, and MSD HSCT for thalassemia were close, at 37% (187 cases), 32% (165 cases), and 30% (156 cases), with another 4 cases of CB HSCT. Regarding autologous transplantation frequencies, those of non-Hodgkin’s lymphoma and multiple myeloma were equivalent. For NHL, 1112 of 1443 patients (77%) chose autologous HSCT, which was much higher than the number choosing allogeneic HSCT including HID (214 cases, 15%), MSD (68 cases, 5%), URD (40 cases, 3%), and cord blood (9 cases) HSCT. Almost all of the multiple myeloma patients received autologous HSCT (1112 of 1136 cases).

Regarding pediatric patients (≤18 years of age), ALL (859 cases, 23%) surpassed AML (849 cases, 22%), accounting for the largest proportion of transplantations, in accordance with its high morbidity among pediatrics. With a proportion of 18%, AA ranked third, followed by thalassemia (15%). Congenital indications, including primary immunodeficiency diseases (PIDs), inherited metabolic disorders (IMDs), and inherited bone marrow failure (IBMF), accounted for a remarkable proportion of 6% altogether, of which 36% were HID, 28% CB, 26% URD, and 10% MSD. Main indications for pediatric patients are shown in Fig. 3c. The main indications for elderly patients were MM (219 cases, 47%), NHL (100 cases, 22%), AML (63 cases, 14%), and MDS (36 cases, 8%) (Fig. 3d).

### Stem cell source

Since nearly all URD (97%) and autologous (99%) HSCTs involved PB as the stem cell source in China, we focused on the stem cell sources of HID and MSD (Table 2). Some centers did not report stem cell source data, so stem cell source data were obtained for 5068 HID (88%) and 1830 MSD (88%) HSCT patients. Single stem cell source of PB or BM accounted for only 30% of HIDs but 72% of MSDs, among which PB was the predominant source, with a proportion of 98%. On the other hand, 70% of HIDs and 28% of MSDs had multiple stem cell sources, which is typical in China. BM + PB HSCT was the main component of HID (59%), and made up 24% of MSDs. 11% of HID and 6% of MSD HSCT used CB as a supplemental component to PB, BM, or PB + BM.

### Conditioning regimen

**Fig. 5:**
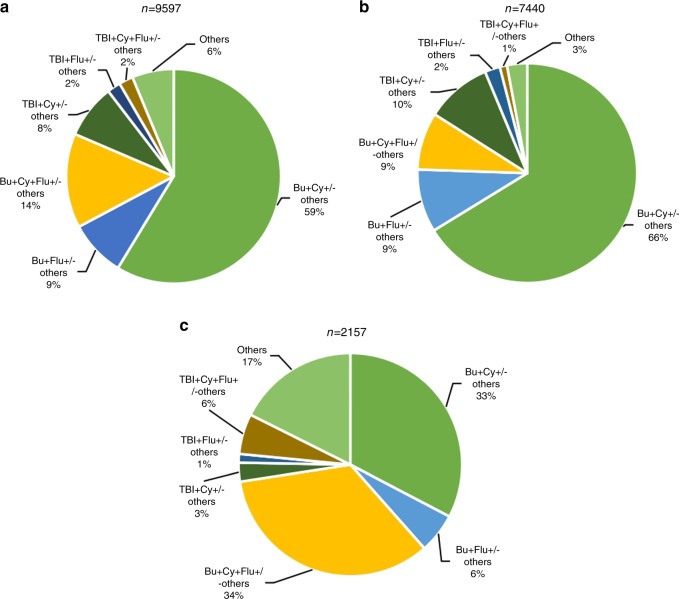
Distribution of conditioning regimen of allogeneic HSCT in China 2019. **a** Relative proportion of conditioning regimen of allogeneic HSCT. **b** Relative proportion of conditioning regimen of malignant disease. **c** Relative proportion of conditioning regimen of non-malignant disease.

As conditioning intensity data were not collected, we could not differentiate reduced-intensity conditioning from myeloablative conditioning, so we paid attention only to the drug composition of the conditioning regimen. As shown in Fig. 5a, for allogeneic HSCT, the BuCy based regimen was the most popular, making up 59% of allogeneic HSCTs. Then came the BuFlu based regimen, accounting for 23% of allogeneic HSCT. For the TBI-based regimen, the proportion was 12%, and among these cases, two-thirds were TBI + Cy-based regimens, and one-third were TBI + Flu-based regimens. HID HSCT, the most frequent type of allogeneic HSCT, had a similar proportion, and ATG was added to 94% of the HID conditioning regimens. As shown in Fig. 5b, among patients with malignant diseases undergoing allogeneic HSCT, the proportion of BuCy based regimen was even larger, reaching 66%. Among malignant diseases, the regimen of lymphoid malignancies (ALL 29% and NHL 25%) had a larger proportion of TBI-based regimens than myeloid malignancies (AML 4% and MDS 3%).In contrast, regimens based on BuFlu constituted 34% of all regimens for nonmalignant disease Fig. 5c), exceeding the BuCy based regimen by 1%. AA, the most momentous non-malignant indication, was performed with BuCy (32%) based regimen most frequently.

For autologous HSCT, the conditioning regimens for MM were based on melphalan (Mel) (79%), BuCy (18%), or other regimens (3%), and those of NHL were based on carmustine/semustine (77%), BuCy (10%), TBI (3%), or other regimens(10%).

## Discussion

This research reflected the current situation and the trend in the rapid development of HSCT in China, with an especially notable increase in HID HSCT. Worldwide, the number of HSCTs has shown sustained growth for decades. The latest report of CIBMTR indicated that the estimated annual number of allogeneic HSCT surpassed 8000 in the US in 2013 and was 9498 in 2019, and the autologous HSCT represents over 60% of HSCTs [9]. Similarly, the number of allogeneic HSCTs in Europe had exceeded 15,000 in 2013 and increased stably to 19798 in 2019, accompanied by 28714 autologous HSCTs [10]. The last report from CBMTRG described a continued growth of transplant activity in China [12]. As the APBMT report has shown, China reported the second largest number of HSCTs in the Asia-Pacific region, accounting for 23% of the total number of HSCT in this region. And China showed the fastest growth in transplant number in this region, with a 2015/2005 ratio of 8.8 [11]. In 2019, the total number of HSCTs in China reached more than 10000 for the first time, with only 22% being autologous, which was different from the numbers in US and Europe but similar to the distribution of the Asia-Pacific region. In this region, Hong Kong and Pakistan reported the smallest proportions of autologous HSCTs, and Japan, Korea, India, Iran, Singapore, Vietnam, Philippines, and Sri Lanka all reported frequencies of autologous HSCTs of less than 50% among all HSCT.

The global increase in the HSCT number has many causes, which may also apply to China. First, the dramatic development of HID HSCT has introduced an era, in which every patient has a donor, especially in China where the family planning policy had led to a lack of MSDs. Second, the disease indications for HSCT have expanded to include more non-malignant diseases and more early-stage malignant diseases. Third, the technological development of HSCT, such as the RIC conditioning regimen, has led to more elderly or complicated recipients receiving HSCT. In addition, the growth of HSCT in our report was partially due to an increase in participating centers.

The number of HID HSCT procedures have increased rapidly worldwide in recent years. Data from CIBMTR show that the number of transplants using HLA-mismatched relative donors surpassed UCB transplants in the US in 2014. The trend has continued to rise, with these transplants representing 21% of allogeneic HSCTs and nearing the number of MSD transplants (23%) in 2019 [9]. The number of HID HSCTs in Europe was 3538 in 2019, an increase of 11.2% from that number in 2018 [10]. However, URD and MSD HSCTs are still the most commong donor types in the US and Europe. In China the number of HID HSCTs surpassed that of MSD HSCTs in 2013, and reached over 5000 for the first time in 2019, representing 60% of allogeneic HSCTs.

MSD HSCT is still the preferred choice for allo-HSCT in China, but the lack of availability has restricted its use. When alternative donors are considered, several factors may affect the choice. The first factor is availability. Then the condition of the recipient (refractory or relapsed status, age, and performance status), characteristics of the alternative donors, and the experience of the transplantation center are considered. Several limitations have decreased the use of URD in China: the probability of finding an appropriate URD is only 11%; the preparation of a MUD would require 3–6 months; and when the patient needs lymphocytes or stem cells for further therapy, re-donation from the URD has little chance of efficacy.

Several HID HSCT modalities, including protocols with T-cell depletion, unmanipulated granulocyte colony-stimulating factor (G-CSF)-primed bone marrow and peripheral blood graft plus anti-thymocyte globulin (ATG)-based regimens, and T-cell replete transplants with post-transplant high-dose cyclophosphamide (PTCY), have been established. The PTCY protocol represented over 90% of HID HSCTs in the US in 2018, and plays a prominent role in Europe. In contrast, the G-CSF/ATG-based modality, also called the “Beijing Protocol”, makes up 94% of HID HSCTs in China. In addition, the use of multiple stem cell source, mainly BM + PB, has become a specific characteristic of HID HSCT in China. However, the main stem cell source is PB in both the US and Europe, although the original studies describing PTCY-based protocols used mainly BM. In the APBMT survey, China reported a rate of 95% of multiple stem cell source for related donor HSCT in 2015.

The Beijing Protocol was established and enriched by the group from Peking University. Wang et al. [13] reported long-term follow-up of HID HSCT for the treatment of leukemia. Of the 756 patients in the study, 99% achieved sustained and full donor chimerism, the 2-year cumulative NRM was 18%, and the 3-year leukemia-free survival rates were 68% and 49% in the standard-risk and high-risk groups respectively. Further studies have shown that the ‘Beijing Protocol’ in HID can provide comparable outcomes to MSD or URD HSCT in both benign diseases and hematologic malignancies [12]. Although PB has become the predominant source of stem cells for HID HSCT in the US and Europe, there is inadequate information about their role compared with bone marrow (BM). A meta-analysis [14] reviewing BM versus PB as a graft source for HID HSCT in adults using PTCY showed significantly higher incidences of grade III-IV acute GVHD (OR = 1.741, 95%CI 1.032–2.938), and grade II-IV acute GVHD (OR = 1.778, 95%CI 1.314, 2.406) and a higher engraftment rate (OR = 1.843, 95%CI 1.066–3.185) in the PB group, with no significant differences were found for relapse incidence, 2-year OS or 2-year DFS between PBSCT and BMT. The G-CSF/ATG based HID HSCT using a combination of PB and BM as a graft source has achieved outcomes comparable to those of MSD HSCT for the treatment of acute leukemia, myelodysplastic syndrome, and severe aplastic anemia [12]. The T cell immune tolerance was proven partially because of G-CSF primed PBST and BM. In addition, Xu et al. [15] demonstrated the priority of G-CSF primed BM + PB to G-CSF primed PB HID HSCT for the treatment of acute leukemia. The BM + PB group achieved higher cumulative myeloid engraftment at 30 days after transplant (89.9 ± 10.1% vs 100%; *P* = 0.04), a lower incidence of 2-year nonleukemic mortality (62.5 ± 14.8% vs 35.1 ± 5.1%; *P* = 0.014), and higher rates of OS (26.8 ± 12.3% vs 43.2 ± 5.0%; *P* = 0.052) and DFS (26.8 ± 12.3% vs 42.4 ± 5.0%; *P* = 0.071) than the PB group. Further clinical trials are needed to confirm the prioritization of the combination of BM + PB graft in other circumstances.

The disease indication has been expanding in both China and other regions in the world, especially for the maturity of transplant technology for non-malignant diseases, among which AA plays the most important role. Data from the CIBMTR show that among 1156 patients receiving MSD HSCT for AA between 2008 and 2018, the 3-year probability of survival was 93% ± 1% for those younger than 18 years and 81% ± 2% for patients 18 years of age or older. Among 1161 recipients of URD HCT during the same period, the probabilities of survival were 85% ± 2% and 74% ± 2% for AA patients under 18 years and ≥18 years respectively [9]. AA had become the third-largest indication for HSCT in China, and more than half received HID HSCT in 2019. In 2012, Xu et al. first reported haplo-SCT with the “Beijing Protocol”, including Bu/Cy and ATG for SAA. All patients achieved 100% donor myeloid engraftment, with a 5-year OS of 68.4% [16]. In a multicenter study, HID and MSD HSCT demonstrated comparable failure-free survival both as salvage treatments and as upfront treatments (86.8% vs. 80.3% and 85.0% vs. 89.8%) [17, 18]. Even for older patients aged 40 years and older, HID might be a feasible alternative option, with comparable OS and FFS rates among HID, MSD, and URD (86.7%, 92.1, 100% and 86.7%, 92.1%, 87.5%) in another study [19].

The use of HSCT in given inherited diseases has been developing. The EBMT reported that in 2019 in Europe, among 19630 allogeneic HSCTs, 4% were primary immune deficiencies and 0.9% were inherited disorders of metabolism [10]. In China, the proportions of the two groups of diseases were both 1% in 2019, and are still in progression. Wang et al. used busulfan-based myeloablative regimen in 34 pediatric cases of mucopolysaccharidosis. The OS at 3 years was 84.8% ± 6.3% and 91.2% of the patients achieved full donor chimerism [20]. HID HSCT has become a useful method for patients with specific inherited disorders of metabolism who lack of matched donors. Chen et al. reported four cases of adrenoleukodystrophy and two cases of mucopolysaccharidosis patient receiving HID HSCT with busulfan, fludarabine, and cyclophosphamide conditioning. All six patients achieved stable engraftment and all were alive after a median follow-up of 292 days [21].

More participating centers have partially contributed to the growth of HSCT reported in China. The participating teams increased by nearly twofold from 76 in 2016 to 149 in 2019. The number of large teams and small teams are both increasing. The number of HSCTs in the largest team has surpassed 1000, and 36% of the teams were small in sized with an annual HSCT number less than 25. This distribution provides both experienced technology and convenient accessibility for eligible HSCT recipients, which may play a more important role in 2020 than ever, when cities are locked down because of COVID-19.

The study has some limitations. Some significant factors, such as the disease status before transplant, the number of transplant, and the transplant outcomes, were not included in this survey. Nevertheless, this study did reveal the current status of HSCT in China, and might provide useful information for health care. Moreover, the data could be the basis for multicenter retrospective studies. To create a complete data pool, we should develop stable method to collect detailed data from all transplant centers in China.

## Supplementary information

Participating teams in the CMBTRG
