# Supplementary material for: Hematopoietic stem cell transplantation activity in China 2019: a report from the Chinese Blood and Marrow Transplantation Registry Group
Source: Bone Marrow Transplant. 2021 Aug 25;56(12):2940–7. doi: 10.1038/s41409-021-01431-6 (PMC8385702; doi:10.1038/s41409-021-01431-6)
Supplement: Supplementary file 1 — Participating teams in the CMBTRG [file 41409_2021_1431_MOESM1_ESM.pdf]

The name list of HSCT team

Peking University Institute of Hematology
The First Affiliated Hospital of Soochow University
Hebei Yanda Lu Daopei Hospital
Anhui Provincial Hospital
Beijing Lu Daopei Hospital
Nanfang Hospital
Blood Diseases Hospital, Chinese Academy of Medical Sciences
Xinqiao Hospital Army Medical University (Third Military Medical University)
Henan Cancer Hospital
Shanghai General Hospital
Fujian Medical University Union Hospital
The First Affiliated Hospital of Zhejiang University School of Medicine
The First Affiliated Hospital of Guangxi Medical University
The First Affiliated Hospital of Zhengzhou University
Shanghai Children's Medical Center
Nanfang-Chunfu Children's Institute of Hematology & Oncology
Wuhan Union Hospital
Aerospace Center Hospital
Chinese PLA General Hospital
Zhujiang Hospital of Southern Medical University
Changhai Hospital
West China Hospital Sichuan University
Children's Hospital of Soochow University
Xiangya Hospital Central South University
Beijing Jingdu Children's Hospital
Shenzhen Children's Hospital
Guangzhou First People's Hospital
The affiliated Hospital of Guizhou Medical University
Guangzhou Women and Children's Medical center
Nanfang Hospital(Department of Pediatrics)
The First Affiliated Hospital of Xi'an Jiaotong University
The First Affiliated Hospital of Sun Yat-sen University
The First Affiliated Hospital of Wenzhou Medical University
Fifth Medical Center of PLA General Hospital
Tangdu Hospital
The First Hospital of Jilin University
The First Affiliated Hospital of Chongqing Medical University
The First Affiliated Hospital of Xinjiang Medical University
Zhejiang Provincial Hospital of TCM
Beijing Children's Hospital
PLA 960th Hospital
The Third Xiangya Hospital of Central South University
Zhejiang Provincial People's Hospital
Shandong Provincial Qianfoshan Hospital
Peking University First Hospital
The Second Hospital of Hebei Medical University
Ruijin Hospital Shanghai Jiao Tong University School of Medicine
Air Force General Hospital, PLA
Forth Medical Center of PLA General Hospital
Shanghai Changzheng Hospital
The Affiliated Hospital of Xuzhou Medical University
Beijing Hospital

Beijing Cancer Hospital
PLA Joint Logistic Support Unit NO.920 Hospital
The First Affiliated Hospital of Harbin Medical University
Nanjing Drum Tower Hospital,The Affiliated Hospital of Nanjing University Medical S[illegible]
West China Second University Hospital, Sichuan University
General Hospital of Western Theater Command
Beijing Chao-yang Hospital
Shandong Provincial Hospital
The second Hospital of Dalian Medical University
No. 940 Hospital of the PLA Joint Logistics Support Force
People's Hospital of Xinjiang Uygur Autonomous Region
Henan Provincial People's Hospital
The Affiliated Hospital of Qingdao University
First People's Hospital of Yunnan Province
Beijing Friendship Hospital, Capital Medical University
Ningbo First Hospital
Guangdong Provincial People's Hospital
Sun Yat-Sen Memorial Hospital, Sun Yat-Sen University
Peking University Third Hospital
The First Hospital Affiliated to Army Medical University
Yantai Yuhuangding Hospital
Guangdong Provincial Hospital of Chinese Medicine
Xinhua Hospital Affiliated to Shanghai Jiao Tong University School of Medicine
Qilu Hospital of Shandong University
The Second Affiliated Hospital of Shanxi Medical University
The First Affiliated Hospital of Xiamen University
The Third Affiliated Hospital of Sun Yat-Sen University
Zhongda Hospital Southeast University
Shengjing Hospital of China Medical University
The Second Affiliated hospital of Zhejiang University School of Medicine
Ningbo Yinzhou People's Hospital
General Hospital of Southern Theater Command
Seventh Medical Center of PLA General Hospital
Shenzhen Second People's Hospital
The First Affiliated Hospital of Anhui Medical University
General Hospital of Northern Theater Command
Renji Hospital Affiliated to Shanghai Jiaotong University School of Medicine
The Sixth Medical Center of PLA General Hospital(Department of Pediatrics)
Affiliated Hospital of Guangdong Medical University
The First Affiliared Hospital of Jinan University
The First Affiliated Hospital of Nanchang University
The First Affiliated Hospital of Guangzhou Medical University
Peking University International Hospital
The First Affiliated Hospital of Henan University of Science and Technology
Affiliated Hospital of Jining Medical University
Liaocheng People's Hospital
Sir Run Run Shaw Hospital, Zhejiang University School of Medicine
Peking University Shenzhen Hospital
Hainan General Hospital
Children's Hospital of Nanjing Medical University
Shanxi Provincial Cancer Hospital
North China University of Science and Technology Affiliated Hospital
Zhongnan Hospital of Wuhan University
The First Hospital of China Medical University

Qingdao Central Hospital
Children's Hospital of Shanghai
The First Affiliated Hospital of Dalian Medical University
Shunde Hospital of Southern Medical University
Southernwest Hospital
Lanzhou University Second Hospital
Weifang People's Hospital
The Fourth Affiliated Hospital of Anhui Medical University
The Fifth Medical Center of PLA General Hospital
Yijishan Hospital of Wannan Medical College
Luoyang Central Hospital Affiliated to Zhengzhou University
Affiliated Hospital of Guilin Medical University
Shanxi Bethune Hospital
First Hospital of Shanxi Medical University
Sun Yat-Sen Hospital affiliated to Sun Yat-Sen University
The First Affiliated Hospital of Zhejiang University School of Medicine (Department
Zhangzhou Affiliated Hospital of Fujian Medical University
PLA Rocket Force Characteristic Medical Center
Jinhua Hospital of Zhejiang University
Linyi People's Hospital
Shandong Provincial Hospital of TCM
Harbin Institute of Hematology & Oncology
Jinhua People's Hospital
The Third People's Hospital of Zhengzhou
Affiliated Zhongshan Hospital of Dalian University
Affiliated Hangzhou First People's Hospital, Zhejiang University School of Medicine
Nanyang Central Hospital
Shulan Hospital
Cangzhou Central Hospital
Central People's Hospital of Zhanjiang
The Second Hospital of Anhui Medical University
The Sixth Medical Center of PLA General Hospital(Department of Hematology)
Tongde Hospital of Zhejiang Province
The Fifth Affiliated Hospital of Sun Yat-Sen University
Zhongshan People's Hospital
Ningbo Yinzhou No. 2 Hospital
Linyi Central Hospital
Heping Hospital Affiliated to Changzhi Medical College
The Second People's Hospital of Linyi
Jiangxi Provincial People's Hospital
People's Hospital of Rizhao
Changzhi People's Hospital
Xi'an Gaoxin Hospital

chool

of Hematology)
